# Cytotoxicity and Genotoxicity of Calcium Hydroxide and Two Antibiotic Pastes on Human Stem Cells of The Apical Papilla

**DOI:** 10.14744/eej.2021.97658

**Published:** 2021-11-16

**Authors:** Davoud JAMSHIDI, Mohamadreza ANSARI, Nematollah GHEIBI

**Affiliations:** 1. Department of Endodontics, Dental Caries Prevention Research Center, Qazvin University of Medical Sciences, Qazvin, Iran; Department of Endodontics, Faculty of Dentistry, Qazvin University of Medical Sciences, Qazvin, Iran; 2. Department of Endodontics, Faculty of Dentistry, Zanjan University of Medical Sciences, Zanjan, Iran; 3. Cellular and Molecular Research Center, Qazvin University of Medical Sciences, Qazvin, Iran

**Keywords:** Calcium hydroxide, cytotoxicity, double antibiotic paste, genotoxicity, triple antibiotic paste

## Abstract

**Objective:**

This study aimed to assess the cytotoxicity and genotoxicity of triple antibiotic paste, double antibiotic paste and calcium hydroxide medicaments on human stem cells of the apical papilla.

**Methods:**

In this experimental study, stem cells were isolated from the apical papilla and cultured. They are treated with different concentrations 0.1, 0.5, 1, 10 and 100 mg/ml of medicaments for 24, 48 and 72 hours. The cytotoxicity and genotoxicity of the medicaments were determined using methyl thiazolyl tetrazolium assay and Comet test, respectively.

**Results:**

Results showed all tested concentrations of the calcium hydroxide had no significant effect on stem cells at any time point. Triple antibiotic paste showed cytotoxicity in 10 and 100 mg/mL concentrations at all-time points and in 1, 10 and 100 mg/ml concentrations at 72 hours. In addition, its genotoxicity was significantly higher than that of other groups (P<0.05). Double antibiotic paste showed cytotoxic effects only in 100 mg/ml concentration at 24 hours and 10 and 100 mg/ml concentrations at 48 and 72 hours. And also, its genotoxicity in these concentrations was significantly higher than that of control and calcium hydroxide groups (P<0.05).

**Conclusion:**

In contrast to calcium hydroxide, triple antibiotic paste and double antibiotic paste, especially in their higher concentrations, induced cytotoxicity and genotoxicity on human stem cells of the apical papilla.

## Introduction

Management of immature teeth with necrotic pulp is often challenging. Due to the presence of an open apex, the application of root filling materials can be difficult. In addition, as the root canal walls can be thin, the mechanical preparation is usually minimised to preserve the dentine, increasing the focus on chemical disinfection (1).

**Highlights**

• TAP induced genotoxicity on SCAPs in all experimental concentrations and timetables, and its cytotoxicity increased in a concentration- and time-dependent manner.
• DAP generated genotoxic and cytotoxic effects on SCAPs in higher concentrations.
• CH had no cytotoxicity and genotoxicity effects on SCAPs in all experimental concentrations and time points.

Regenerative endodontics is defined as ‘biologically based procedures designed to replace damaged tooth structures with the pulp-dentine complex’ (2). Its ultimate goal is to replace the affected or necrotic pulp tissue with cells of pulp-dentine complex in order to promote tooth longevity and function (2). The first case report illustrating regenerative endodontic procedures (REPs) resembling current the American Association of Endodontists' guidelines was published in 2001 (3,4). Since then, many case reports have reported signs of improvement of apical periodontitis, the continuation of root development and in some cases, normal response to vitality tests following regenerative endodontic treatments (5). The advantages of this technique include continuation of root development and increase in root length, increase in thickness of dentinal canal walls and closure of the apex (6). However, the cervical region of teeth still have remained susceptible to fracture after REPs (7).

At present, REPs are recommended for the treatment of immature teeth with necrotic pulp (6). However, successful REPs depends on the disinfection of the root canal system (8). Therefore, various irrigants and medicament are applied for root canal disinfection (9). Current REP protocols of the American Association of Endodontists and other studies have been recommended using triple antibiotic paste (TAP), double antibiotic paste (DAP) and calcium hydroxide (CH) as root canal medicaments (4,10).

The apical papilla contains different cell types, especially stem cells of the apical papilla (SCAPs) that are proposed to play a crucial role in root formation (11). An ideal root canal medicament for regenerative endodontics should have broad-spectrum antibacterial activity and maintain SCAPs viability (12). Therefore, any deleterious effects of these medications on the stem cells may hinder the success of REPs. To the best of our knowledge, no previous studies have evaluated the genotoxicity of CH, TAP and DAP on human SCAPs. Previous studies have evaluated the cytotoxicity of these medicaments in other concentrations on different cells (13-17). Considering the lack of information on the effect of time and concentrations on SCAPs’ survival, this study aimed to assess the cytotoxicity and genotoxicity of CH, TAP and DAP as the most commonly used intracanal medicaments against SCAPs.

## Materials and Methods

### Culture of Human SCAPs

The study protocol was peer-reviewed and approved by the Institutional Ethics Committee of Qazvin University of Medical Sciences (Code: IR.QUMS.REC.1396.447).

This experimental study evaluated five impacted immature mandibular third molars, each one from 16 to 19-year-old donors with no systemic disease and no history of maxillofacial surgery. The donors consented to the use of their teeth in this study. The teeth had an indication for extraction, were completely sound, and 2/3 of their roots had been formed as noticed on periapical radiographs. Immediately after extraction, the teeth were rinsed with phosphate-buffered saline (PBS; Gibco BRI, Grand Island, NY, USA) and remained in PBS until SCAP harvesting. The apical papilla tissue was separated from the dental pulp using a sterile scalpel. Stem cells were isolated from the apical papilla by enzymatic digestion using 2 mg/ml of type I collagenase (Worthlington Biomedical, Lakewood, NJ) and immersed in Dulbecco’s modified Eagle’s medium (DMEM; Gibco, Grand Island, NY, USA) in sterile cell culture flasks (SPL Life Science, Gyeonggi-do, South Korea). The cells were then passaged in a culture medium containing 15% fetal bovine serum (Gibco, Grand Island, NY, USA). After 4 passages, adequate cell confluence was reached. This was repeated 5 to 8 times to obtain a large population of cells. The culture medium was refreshed every 2 to 3 days and the cells were passaged again after 1 week.

To confirm the mesenchymal origin of the cultured cells, flow cytometry was performed to determine the percentage of mesenchymal cells by tracing CD90 and CD105 mesenchymal markers (Chemicon, USA) and CD45 hematopoietic marker (Chemicon, USA) (18).

To freeze the cells, cells adhered to the bottom of the flask had to be detached. For this purpose, the overlaying medium was discarded, then the flask containing the cells was rinsed with PBS. The PBS was then discarded, and the flask was trypsinised. After detachment of cells, a complete culture medium was added to the flask, and the contents of the flask were transformed into sterile falcon tubes and centrifuged (Farxane Arman, Iran) at 800 rpm for 7 minutes. The supernatant was discarded and 2 ml of complete culture medium was added to cells deposited at the bottom. The cells were then counted and 1 ml of culture medium was added per 1-2x10^6^ cells for freezing. The cell suspension was then transferred into sterile cryo-tubes and frozen at -20°C for 24 hours. They were then transferred to another freezer at -70°C and from there to a nitrogen tank. For defrosting, the cryo-tubes were incubated, and their contents were transferred to sterile falcon tubes; 3 mL of DMEM was added, and they were centrifuged at 1500 rpm for 3 minutes. The supernatant was discarded and 1 mL of fresh DMEM containing fetal bovine serum was added and incubated.

### Cytotoxicity testing

The methyl thiazolyl tetrazolium (MTT) assay was used to assess cytotoxicity where 50 mg of MTT powder was dissolved in 10 ml of PBS and filtered using 0.22 μm pore sterile filters. DAP was prepared by mixing metronidazole and ciprofloxacin in a 1:1 weight ratio (Sigma Aldrich, USA). TAP was prepared by mixing metronidazole, ciprofloxacin and minocycline in a 1:1:1 weight ratio (Sigma Aldrich, USA) (19). Calcium hydroxide (Master Dent, USA) with 10 mg/mL concentration was dissolved in DMEM and incubated for 72 hours. The culture medium was then filtered, and 0.1, 0.5, 1, 10 and 100 mg/ml concentrations of each material were prepared (13,14). To assess the cytotoxicity of different concentrations of the products, 5000 cells were cultured in each well of a well-plate. The cells were incubated for 24 hours. Their culture medium was then replaced and DAP, TAP and CH in the afore-mentioned concentrations were added to the culture medium. The group receiving standard cell culture medium without treatment samples was considered as the negative control group. Cell viability at all groups was compared with negative control group. The plates were incubated for 24, 48 and 72 hours (15) and then 10 μl of MTT solution and 90 μl of DMEM containing 10% fetal bovine serum were added to each well. They were then incubated at 37°C for 3 hours. After this time period, the formazan crystals were visible in viable cells under an inverted microscope (MBL3200, Germany). The overlaying medium in each well was then gently removed and 100 μl of dimethyl sulfoxide was added to each well. After several pipetting of each well in order for the formazan crystals to dissolve, the optical density was measured using an ELISA reader (ELX800; BioTek, USA) at 540-690 nm wavelengths.

The experiments were performed in triplicate.

### Genotoxicity testing

An alkaline Comet test was used for the assessment of genotoxicity (20,21). For this purpose, the cells were first treated with 0.1 mg/ml concentration of the respective materials for 24 hours (22). They were then trypsinised and 200 μL of cell suspension was mixed with 200 μl of agarose gel with low melting temperature (1%). Next, 300 μl of cell suspension was applied on one side of a slide covered with the conventional agarose gel and spread with a coverslip. The slides were refrigerated for the gel to set. After 5 to 10 minutes, the lysis solution was added and the slides were refrigerated again for 1 hour. They were then placed in an electrophoresis tank (BV-104, Germany), and the denaturing solution was poured on the slides. Next, the tank was covered with aluminium wrap. A 30 minute period allowed DNA strands to uncoil. The tank was then connected to electric power (1 v/cm), and the slides remained in this condition for 30 minutes. The slides were then placed in a neutralising buffer for 5 minutes and stained with 20 μg/μl of ethidium bromide (Sigma Aldrich, USA). Next, the samples were photographed under a fluorescence microscope (LW Scientific, USA), and the results were analysed using the Comet Score IV software.

### Image analysis

An analysable image by Comet Score IV software included 1 cell with 2 detectable regions of head and tail. In addition, the following parameters were assessed for quantitative analysis of the frequency of DNA breaks:

Tail length or the percentage of DNA present in the Comet tail: The magnitude of movement of DNA segments from the central region shows the frequency of breaks.

Olive tail moment: Tail moment is the product of tail length and a fraction of the entire DNA in the tail. The final moment includes the smallest size of detectable DNA and the number of fractured segments.

Olive Tail Moment=(Tail. mean - Head. mean) × Tail %DNA/100.

Head % DNA=(Head Intensity/(Head Intensity + Tail. Intensity)) × 100.

Tail % DNA=100 – Head %DNA

### Statistical analysis

Normal distribution of data was confirmed using the Shapiro Wilk test. Levene’s test confirmed the homogeneity of variances (P=0.410). In addition, the mean and standard error values were reported. Data were analyzed using two-way ANOVA at a 0.05 level of significance.

## Results

### Isolation and culture of SCAPs

The cells showed a spindle shape and created a homogenous population after four passages.

### Confirming the mesenchymal origin of cultured cells using cell surface markers

Evaluation of cell surface markers confirmed the mesenchymal origin of cells. Of all cells, 89.92% expressed CD90 and 84.50% expressed CD105 marker on their surface. They were negative for hematopoietic markers. For example, only 7.62% of cells expressed CD45 on their surface.

### Results of cytotoxicity assessment Effect of DAP on SCAPs

**Figure 1.**
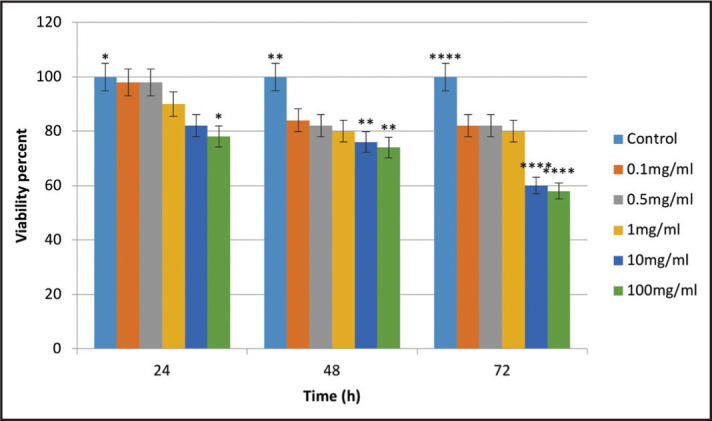
Cytotoxicity comparison of different concentrations of DAP with the control group on SCAP viability at different time points (*P<0.05, **: P<0. 01, ****: P<0.0001)

The results showed that over time (from 24 to 72 hours), the percentage of viable cells exposed to different concentrations of DAP significantly decreased (P<0.05), but the percentage of viable cells was not significantly different when different concentrations of DAP were compared (P>0.05). At 24 hours, a significant difference was noted in cell viability between 100 mg/ml concentration of DAP and the control group (P<0.05). At 48 and 72 hours, significant differences were noted in cell viability between 10 and 100 mg/ml concentrations of DAP and the control group (P<0.05) (Fig. 1).

### Effect of TAP on SCAPs

**Figure 2.**
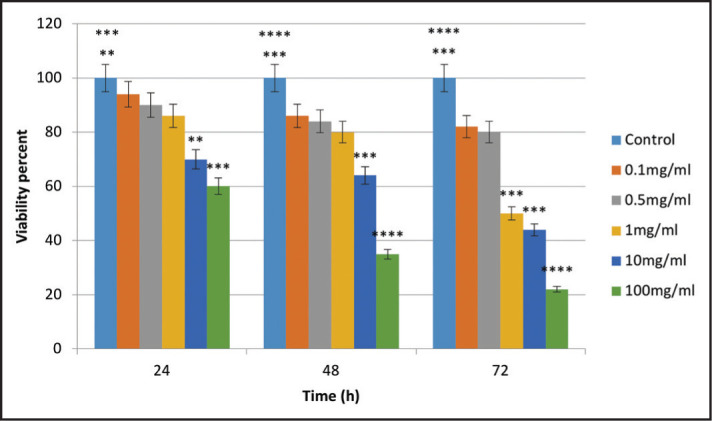
Cytotoxicity comparison of different concentrations of TAP with the control group on SCAP viability at different time points (**P<0.01, ***P<0.001, ****P<0.0001)

The results showed that as the concentration of TAP and time increased, the percentage of cell viability decreased. The difference in reduction between different concentrations was significant at all-time points (P<0.05). Significant differences were noted between 10 and 100 mg/ml TAP and the control group at 24 and 48 hours, and 1, 10 and 100 mg/ml concentrations of TAP and the control group at 72 hours (Fig. 2).

### Effect of CH on SCAPs

**Figure 3.**
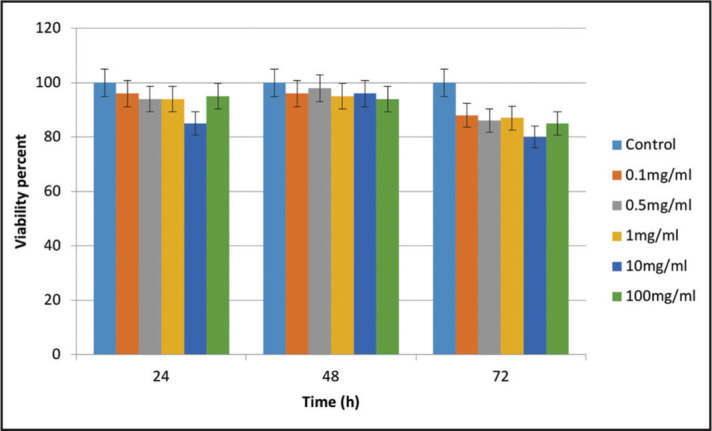
Cytotoxicity comparison of different concentrations of CH with the control group on SCAP viability at different time points

The results showed that by an increase in time and concentration of CH, no change occurred in cell viability (P>0.05) (Fig. 3).

### Results of Comet test for genotoxicity assessment

**Figure 4.**
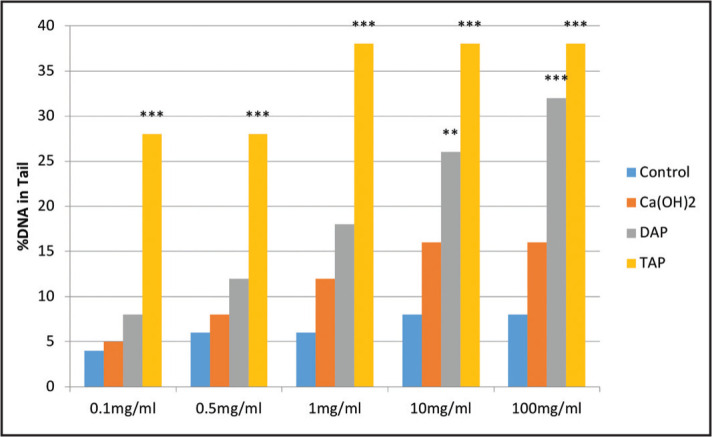
Percentage of DNA present in Comet tail of SCAPs in the control group and different concentrations of TAP, DAP and CH at 24 hours (**p≤0.01, ***p≤0.001)

**Figure 5.**
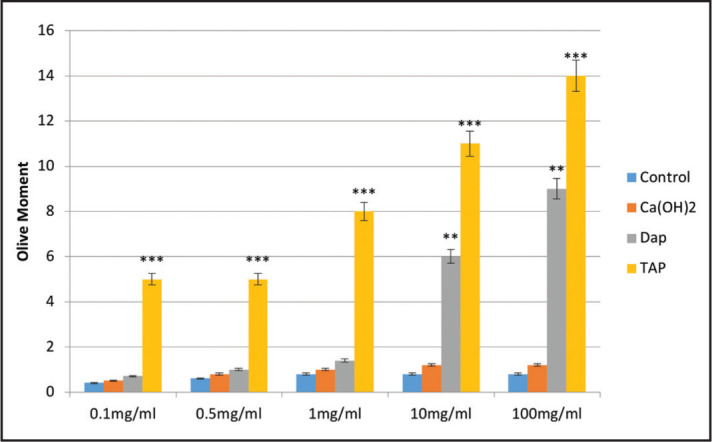
Olive moment factor of Comet tail of SCAPs in the control group and different concentrations of TAP, DAP and CH at 24 hours (**P≤0.01, ***P≤0.001)

Ten cells in each sample were analysed. Figure 4 shows the percentage of DNA present in Comet tail, and Figure 5 shows the olive moment factor. The percentage of DNA in the Comet tail of SCAPs increased in the test groups compared with the control group. This increase in all concentrations of TAP was significant compared with the control group. The increase in 10 and 100 mg/ml concentrations of DAP was also significant compared with the control group.

The olive moment factor in the Comet tail of SCAPs increased in the test compared to the control group. This increase in all concentrations of TAP was significant compared with the control group. Also, this increase in 10 and 100 mg/ml concentrations of DAP was significant compared to the control group.

## Discussion

This study assessed the cytotoxicity and genotoxicity of TAP, DAP and CH intracanal medicaments on SCAPs. The cell surface markers were used for confirming the mesenchymal origin of cultured cells. Several studies documented that cells that expressed CD90 and CD105 and showed a lack of CD45 are considered as mesenchymal stem cells (23, 24).

The results showed that DAP and TAP decreased the viability of SCAPs in a concentration and time-dependent manner. However, significant differences were obtained at 100 mg/mL concentration of DAP at 24 hours treatment and 10 and 100 mg/mL concentrations at 48 and 72 hours treatment compared to the control group. Also, TAP decreased the cell viability of SCAPs significantly in all treatment concentrations and times scales compared to control. These findings highlighted the cytotoxicity of DAP and TAP, which at their higher concentrations show a good agreement with the results of Althumairy et al. (13), indicating that TAP and DAP in high concentrations (1000 mg/ml) had cytotoxic effects on SCAPs, but were safe for cells in 1 mg/ml concentration. In another study, Chuensombat et al. (15) showed that TAP had dose- and time-dependent cytotoxicity for dental pulp stem cells and SCAPs. Ruparel et al. (16) demonstrated that both TAP and DAP decreased the viability of SCAPs in a dose-dependent manner and had no adverse effect on cell viability in concentrations lower than 0.1 mg/ml. Our results revealed that DAP at concentrations lower than 10 mg/mL did not induce toxicity on SCAPs, but TAP showed the toxic effect on SCAPs survival at 1 mg/ml. Similar to this study, Labban et al. (14) assessed the direct cytotoxic effects of TAP and DAP on stem cells of the dental papilla and emphasizes to the highest cytotoxicity of TAP in 5 mg/ml concentration. DAP and TAP in 0.3 and 2 mg/ml concentrations had no adverse effect on cell proliferation. Although, in this study, increased concentration of TAP and DAP and duration of exposure significantly decreased cell viability of SCAPs, while this was not the case for CH.

In line with our results, Yadlapati et al. (17) reported that TAP and minocycline had higher cytotoxic effects on human gingival fibroblasts compared to DAP and CH. It has been shown that the cytotoxic effect of a mixture of metronidazole, ciprofloxacin and minocycline was higher than that of each antibiotic alone, and 0.39 mg/ml concentration had the lowest cytotoxicity (15). Similarly, our study showed higher cytotoxicity of TAP compared to DAP against SCAPs; in other words, by an increase in the number of antibiotics in the mixture, the cytotoxicity of the mixture was increased. Furthermore, the higher cytotoxicity of TAP compared with DAP can be due to the presence of minocycline in its composition (15,17). In general, variability in the results of studies can be due to different antibiotic manufacturers, different preparation techniques and different cell viability assays used (15). Fahmy et al. (25) showed revascularization potential of DAP was significantly better than modified TAP.

The acidic pH of antibiotics can also explain their cytotoxicity, which directly affects cells and increases the absorption of other materials (26,27). To eliminate this problem, we used pure antibiotic powders in our study. 

In line with other studies (13,16), our results showed no significant change of the CH on cell viability with increased concentration or treatment time. These studies also showed that CH had no adverse effect on the viability of SCAPs and significantly increased their proliferation in 1 mg/ml concentration. Low concentrations of CH upregulate the phosphorylated extracellular signal-related kinases, which is a marker for the proliferation of dental pulp and periodontal ligament stem cells (28). Kitikuson et al. (11) also showed TAP-treated dentine had negative effects on the SCAPs attachment. In contrast, CH-medicated dentine had no detrimental effect on SCAPs attachment. It seems that the CH residues that remain after rinsing positively affect the SCAPs (13). Also, CH induces the release of tumour growth factor beta-1 from dentine (29), which has a proliferative effect on mesenchymal stem cells (30). Selis et al. (12) mentioned CH as the gold standard in intracanal medicament in the practice of endodontics.

This is the first study to assess the genotoxicity of DAP and TAP on SCAPs, which is a strength of this study. Genocytoxic materials are those with affinity to interact with DNA and cause potentially mutations and cancer (31). The results showed that the genotoxicity and cytotoxicity of TAP in all concentrations were significantly higher than that of other materials, which may be due to the presence of minocycline in its composition (15,17). However, no previous study is available in this regard to compare our genotoxicity results with it. Furthermore, the genotoxicity of DAP in 10 and 100 mg/ml concentrations was significantly higher than that of control and CH groups, which may indicate that in addition to the type of antibiotic, its high concentration can also play a role in genotoxicity.

It was shown that DNA damage affects the survival and function of the stem cells and may threaten their tissue regeneration potential (32).

Further studies using other methods to assess genotoxicity and cytotoxicity are also required to confirm the current findings. Moreover, future studies are required to find the most efficient concentration of antibiotics with the lowest cytotoxic and genotoxic effects for regenerative treatments. Finally, animal studies are eventually required to test the accuracy of findings *in vivo*.

## Conclusion

Within the limitations of this *in vitro* study, the results showed that CH had no significant effect on SCAPs survival, but TAP and DAP induced both cytotoxic and genotoxic effects on SCAPs after 72 h exposure. The TAP showed highest deleterious effects on stem cells viability in all treated concentrations and time in comparison to other groups. Thus, selecting the type and concentration of intracanal medicaments, especially antibiotics, should be chosen with caution in REPs.

### Disclosures

**Conflict of interest:** None.

**Ethics Committee Approval:** This study was approved in the ethics committee of Qazvin University of Medical Sciences (IR.QUMS.REC.1396.447).

**Peer-review:** Externally peer-reviewed.

**Financial Disclosure:** None.

**Authorship contributions:** Concept – D.J.; Design – D.J., M.A.; Supervision – D.J.; Funding - None; Materials - M.A.; Data collection &/or processing – M.A.; Analysis and/or interpretation – M.A., N.G.; Literature search – D.J., N.G.; Writing – D.J., M.A.; Critical Review – D.J., M.A., N.G.
